# Patronize Home Industry

**Published:** 1887-12

**Authors:** 


PATRONIZE HOME INDUSTRY.—We say, with confidence, and without fear of successful contradiction, for we speak from the book, and from experience, that the Steam Job and Book Printing House of Eugene von Boeckmann, in Austin, can not be excelled in their line of work ; and as for prices, we will guarantee that they can compete with St. Louis and Chicago. The Transactions of the Texas State Medical Association was printed by this house, (lowest and best bid), and it has received complimentary mention by the leading journals throughout the United States. The printing of this JOURNAL has been done by them from the beginning, and is eminently satisfactory as to style and price. *Physicians' bill heads, envelopes, prescription blanks, etc.*, will receive *especial attention* and sent C.O.D. Orders solicited and satisfaction guaranteed.

